# Bundle sheath chloroplast volume can house sufficient Rubisco to avoid limiting C_4_ photosynthesis during chilling

**DOI:** 10.1093/jxb/ery345

**Published:** 2018-11-08

**Authors:** Charles P Pignon, Marjorie R Lundgren, Colin P Osborne, Stephen P Long

**Affiliations:** 1 University of Illinois, Carl R. Woese Institute for Genomic Biology and Departments of Crop Sciences and of Plant Biology, Urbana, IL, USA; 2 Department of Civil and Environmental Engineering, Massachusetts Institute of Technology, Cambridge, USA; 3 Arnold Arboretum, Harvard University, Boston, USA; 4 Lancaster Environment Centre, Lancaster University, Lancaster, UK; 5 Department of Animal and Plant Sciences, Alfred Denny Building, University of Sheffield, Sheffield, UK

**Keywords:** *Alloteropsis*, bundle sheath, C_4_ photosynthesis, chilling tolerance, chloroplast, cold tolerance, confocal microscopy, maize, *Miscanthus*, sugarcane

## Abstract

C_4_ leaves confine Rubisco to bundle sheath cells. Thus, the size of bundle sheath compartments and the total volume of chloroplasts within them limit the space available for Rubisco. Rubisco activity limits photosynthesis at low temperatures. C_3_ plants counter this limitation by increasing leaf Rubisco content, yet few C_4_ species do the same. Because C_3_ plants usually outperform C_4_ plants in chilling environments, it has been suggested that there is insufficient chloroplast volume available in the bundle sheath of C_4_ leaves to allow such an increase in Rubisco at low temperatures. We investigated this potential limitation by measuring bundle sheath and mesophyll compartment volumes and chloroplast contents, as well as leaf thickness and inter-veinal distance, in three C_4_ Andropogoneae grasses: two crops (*Zea mays* and *Saccharum officinarum*) and a wild, chilling-tolerant grass (*Miscanthus* × *giganteus*). A wild C_4_ Paniceae grass (*Alloteropsis semialata*) was also included. Despite significant structural differences between species, there was no evidence of increased bundle sheath chloroplast volume per leaf area available to the chilling-tolerant species, relative to the chilling-sensitive ones. Maximal theoretical photosynthetic capacity of the leaf far exceeded the photosynthetic rates achieved even at low temperatures. C_4_ bundle sheath cells therefore have the chloroplast volume to house sufficient Rubisco to avoid limiting C_4_ photosynthesis during chilling.

## Teaser

The volume of bundle sheath chloroplasts available for Rubisco investment in the leaves of four C_4_ grasses could potentially support much greater photosynthetic activity than is typically observed, even at chilling temperature.

## Introduction

C_4_ photosynthesis involves a biochemical CO_2_ concentrating mechanism. In mesophyll cells, the enzyme phospho*enol*pyruvate carboxylase assimilates CO_2_ into oxaloacetate, which is then metabolized into further C_4_ compounds that are transferred to, and decarboxylated in, bundle sheath (BS) cells to raise [CO_2_] around the enzyme Rubisco (von Caemmerer and Furbank, 2003). Rubisco then fixes this CO_2_ via the Calvin–Benson cycle in the BS. In C_4_ plants, Rubisco is therefore predominantly localized to the chloroplasts of BS cells, where the increased [CO_2_] greatly improves photosynthetic efficiency because it effectively eliminates photorespiration, the energetically costly process initiated when O_2_ instead of CO is fixed by Rubisco (Hatch, 1987). The BS cells of C_4_ leaves are arranged radially around veins and isolated from internal leaf air spaces by surrounding mesophyll cells (Dengler and Nelson, 1999). Relative to the leaves of C_3_ plants, C_4_ leaves achieve greater overall BS tissue area via a combination of higher vein density, enlarged BS cells, and more numerous BS cells (Christin *et al.*, 2013; Lundgren *et al.*, 2014).

The enhanced efficiency of C_4_ photosynthesis under warm conditions is evident in the high productivity of the Andropogoneae grass crops maize (*Zea mays* L.), sorghum (*Sorghum bicolor* (Lu.) Moench), and sugarcane (*Saccharum officinarum* L). However, photosynthesis in the majority of C_4_ grasses is characterized by poor chilling tolerance, limiting them to warmer environments (Long, 1983; Sage, 2002; Long and Spence, 2013). Improving chilling tolerance could therefore expand the growing region and lengthen the growth seasons of C_4_ crops (Głowacka *et al.*, 2016). Such tolerance of low temperatures has evolved many times in wild C_4_ grasses, enabling them to shift their niches into cooler alpine or temperate environments (Watcharamongkol *et al.*, 2018).

The mechanisms conferring chilling tolerance to C_4_ grasses have been especially well studied in the grass *Miscanthus* × *giganteus* Greef et Deu. because of its importance for cellulosic biomass production (Heaton *et al.*, 2010). For example, *Z. mays* leaves developing at 14 °C have less than 10% of the photosynthetic capacity of *Z. mays* leaves developing at 25 °C, while leaves of *M.* × *giganteus* are unaffected by this temperature difference (Long and Spence, 2013). Another study found that *M.* × *giganteus* achieved 59% greater biomass than *Z. mays* by producing photosynthetically competent leaves earlier in the year and maintaining them several weeks after *Z. mays* senesced in side-by-side trials in the US Corn Belt (Dohleman and Long, 2009). This growth advantage may be even more pronounced in the near future, as anthropogenic climate change may cause more frequent and intense springtime chilling events across the US Corn Belt (Kim *et al.*, 2017). Understanding and harnessing the potential of chilling-tolerant C_4_ photosynthesis could provide crucial improvements to the yield and robustness of key C_4_ crops (Long *et al.*, 2006; Zhu *et al.*, 2010; Yin and Struik, 2017).

Chilling tolerance in C_4_ grasses may be linked to leaf anatomy. Because C_4_ leaves restrict Rubisco to BS cells, the space potentially available to house this enzyme is roughly halved relative to C_3_ leaves, which can accommodate the enzyme in all photosynthetic cells (Pittermann and Sage, 2000). Under moderate temperatures, flux analysis points to Rubisco as a major control point on the rate of CO_2_ assimilation in C_4_ leaves, as it is in C_3_ leaves (Furbank *et al.*, 1997). Since catalytic rate declines with temperature, Rubisco becomes an even greater limitation under chilling, unless its amount is increased (Sage *et al.*, 2011; Long and Spence, 2013).

It has been proposed that BS chloroplast volume would limit acclimatory increases in Rubisco in C_4_ plants at chilling temperatures (<15 °C), so disadvantaging them relative to their C_3_ counterparts (Pittermann and Sage, 2000; Kubien *et al.*, 2003; Kubien and Sage, 2004; Sage and McKown, 2006; Sage *et al.*, 2011). This hypothesis is supported by the observation that leaves of chilling-tolerant C_3_ plants often increase Rubisco content during acclimation, whereas this is rarely seen in C_4_ leaves (Sage and McKown, 2006; Long and Spence, 2013). Net photosynthetic CO_2_ uptake (*A*_sat_) in C_4_ leaves correlates with Rubisco content (Pearcy, 1977) and activity (Pittermann and Sage, 2000; Kubien and Sage, 2004; Friesen and Sage, 2016) at low (<15 °C), but not high (>25 °C), temperatures. Rubisco’s flux control coefficient over photosynthetic CO_2_ assimilation reaches 0.99 (i.e. near-total control) at 6 °C in *Flaveria bidentis* L. Kuntze (Kubien *et al.*, 2003). These observations raise important questions: does Rubisco limit photosynthesis in all C_4_ plants at low temperatures, and is this limitation specifically imposed by the restricted space available in the BS to house the enzyme?

Under chilling conditions, the chilling-tolerant *M.* × *giganteus* maintains photosynthetic capacity and, unusually, maintains or slightly increases leaf Rubisco content per unit leaf area, while showing large increases in pyruvate P_i_ dikinase (PPDK) expression (Naidu *et al.*, 2003; Wang *et al.*, 2008*b*; Long and Spence, 2013). Accessions of *M. sacchariflorus*, one of the parent species of *M.* × *giganteus*, achieved some of the highest light-saturated rates of leaf CO_2_ uptake (*A*_sat_>16 µmol m^−2^ s^−1^) recorded for any plant grown and measured at 15 °C (Głowacka *et al.*, 2015), showing that this species must accumulate sufficient Rubisco to support such high photosynthetic rates. Of course, there is the possibility that these *Miscanthus* genotypes are exceptional in providing unusually large bundle sheath chloroplast volumes.

Coinciding with the acclimation of C_4_ cycle enzymes in *Miscanthus*, the up-regulation of key photoprotective mechanisms reduces damage to photosystem II (Farage *et al.*, 2006). This suggests that decreased photosynthetic rates in most C_4_ grasses at low temperature have multiple causes rather than arising from one inherent limitation. Indeed, comparative transcriptomics has suggested that the chilling tolerance of photosynthesis in *M.* × *giganteus* corresponds to the up-regulation of genes encoding several photosynthetic proteins (Spence *et al.*, 2014). *Miscanthus* × *giganteus* maintains the linear relationship between operating photochemical efficiency of photosystem II and the quantum efficiency of CO_2_ assimilation during chilling, suggesting that the balance of C_3_ and C_4_ cycles is not compromised (Naidu and Long, 2004). In total, these findings suggest that Rubisco is not the sole limitation to C_4_ photosynthesis at chilling temperatures, and that any volume limitation imposed by restriction of the enzyme to the bundle sheath can be overcome, at least in the case of *M.* × *giganteus* and related species (Long and Spence, 2013).

Because most Rubisco in C_4_ leaves is confined to BS chloroplasts, a measure of the total volume of chloroplasts in the BS is required to determine if there is enough space available to increase Rubisco content in C_4_ leaves. However, most attempts at chloroplast quantification have not documented 3D measurements, but rather chloroplast counts and 2D planar area (Pyke and Leech, 1987; Brown and Hattersley, 1989; Stata *et al.*, 2014, 2016). With confocal laser scanning microscopy, it is possible to measure chloroplast volume directly from an optically produced 3D image (Park *et al.*, 2009; Coate *et al.*, 2012). Chloroplast measurements have previously been made on fixed, dehydrated samples in accordance with TEM imaging procedures (Sage and Williams, 1995). While this method is adequate for relative comparisons of chloroplast size and number between plant taxonomic clades or functional types (Stata *et al.*, 2016; Stata *et al.*, 2014), it may distort chloroplast shape and prevent accurate estimation of absolute chloroplast volume *in vivo*. Cryo-sectioning and analysis of fresh plant material may prevent this type of distortion.

To test the hypothesis that BS chloroplast volume restricts the capacity for Rubisco to the extent that it would limit photosynthesis in C_4_ grasses, chloroplast volume and associated leaf anatomical characteristics were measured, and used to calculate the amount and activity of Rubisco that could be supported on a leaf area basis. The focus of the study was on grasses of the Andropogoneae: since *M.* × *giganteus* appears to escape the low temperature limitation observed in most C_4_ grasses, its BS chloroplast volumes were compared to two chilling-intolerant crop species of the same tribe (*Z. mays* and *S. officinarum*). The unrelated, non-Andropogoneae, non-crop and chilling-intolerant C_4_ grass (*Alloteropsis semialata* J. Presl) was also included in the study (Osborne *et al.*, 2008).

## Materials and methods

### Plant material

Measurements were taken on *Z. mays* cv. FR1064, *S. officinarum* hybrid complex cultivar cv. CP88-1762, a C_4_ lineage of *A. semialata* originating from South Africa (Osborne *et al.*, 2008), and the ‘Illinois’ clone of *M.* × *giganteus*. *Miscanthus* × *giganteus* was grown in the field and the other species were grown in a controlled-environment greenhouse, maintained between 25 and 30 °C with high pressure sodium lamps ensuring an average photon flux of 450 μmol m^−2^ s^−1^ over a 12 h photoperiod.

*Miscanthus* × *giganteus* was grown at the University of Illinois Agricultural Research Station farm near Champaign, IL, USA (40°02′N, 88°14′W, 228 m above sea level). Soils at this site are deep Drummer/Flanagan series (a fine silty, mixed, mesic Typic Endoaquoll) with high organic matter typical of the central Illinois Corn Belt. Fertilizer was not applied. As in previous studies, the youngest fully expanded leaf of *M.* × *giganteus* plants, as judged by ligule emergence, was sampled in July (Dohleman *et al.*, 2012; Arundale *et al.*, 2014*a*,b; Pignon *et al.*, 2017).

*Alloteropsis semialata* and *Z. mays* seeds were germinated on moist filter paper in a growth chamber maintained at 25 °C with an average photon flux of 200 μmol m^−2^ s^−1^. They were then transferred to pots of soil-less cultivation medium (LC1 Sunshine Mix, Sun Gro Horticulture, Agawam, MA, USA), with additional coarse sand and perlite mixed into pots for *A. semialata*. Single stem segments of *S. officinarum* were planted directly into pots of a second soil-less cultivation medium (Metromix 900: SunGro Horticulture). All pots were watered daily to field capacity. *Zea mays* was initially fertilized with granulated fertilizer (Osmocote Plus 15/9/12, The Scotts Company LLC, Marysville, OH, USA) followed by general nutrient solution (Peter’s Excel 15-5-15, Everris NA Inc., Dublin, OH, USA) and iron chelate supplement (Sprint 330, BASF Corp. NC, USA) added to the watering regime once every week. *Alloteropsis semialata* and *S. officinarum* were fertilized with granulated fertilizer (Osmocote Classic 13/13/13, The Scotts Company LLC), and *A. semialata* supplemented with iron chelate (Sprint 330, BASF Corp.). Plants were grown until at least the fifth leaf was fully expanded, as judged by ligule emergence, and the youngest fully expanded leaf was sampled.

### Sample preparation and measurement

On sampling, leaves were immediately immersed in a glycol and resin based cryostat embedding medium (Tissue-Tek O.C.T. Compound, Sakura Finetek, Torrance, CA, USA), which provides solid sectioning support on dry ice. Transverse sections of 40 µm were cut (Leica CM3050 S, Leica Biosystems, Wetzlar, Germany) and mounted on glass slides. Slides were then immersed for 15 min in a cell membrane and wall dye solution (FM 1-43FX, Thermo Fisher Scientific, Waltham, MA, USA), and diluted to 3.6 mM in dimethylsulfoxide (Thermo Fisher Scientific) and water, in order to image cell walls. Samples were imaged with a confocal laser-scanning microscope (LSM 700, Carl Zeiss AG, Oberkochen, Germany). Images were acquired through a ×63 oil-immersion objective (×63 Plan-Apochromat, Carl Zeiss AG) for *M.* × *giganteus*. It was determined that reduced magnification could be used to widen the field of view while still providing accurate estimates of chloroplast volume. Therefore a ×40 oil-immersion objective (×40 Plan-Apochromat, Carl Zeiss AG) was used for *Z. mays*, *S. officinarum*, and *A. semialata*.

The fluorescence of dye-labelled cell walls was analysed by excitation at 555 nm, and emission was detected at a bandpass of 405–630 nm. Chlorophyll was excited at 633 nm, and its fluorescence emission was detected at a bandpass of 630–700 nm. Serial optical sections were obtained at 1-µm depth intervals, i.e. in the *z*-axis (Zen software, Carl Zeiss AG). Although sampling depth (8–15 µm in the *z*-axis) was insufficient to capture whole BS cells, each leaf section contained a random sampling of cells, which avoided the risk of biasing measurements due to non-homogeneous chloroplast distribution through the length of the cell.

**Fig. 1.**
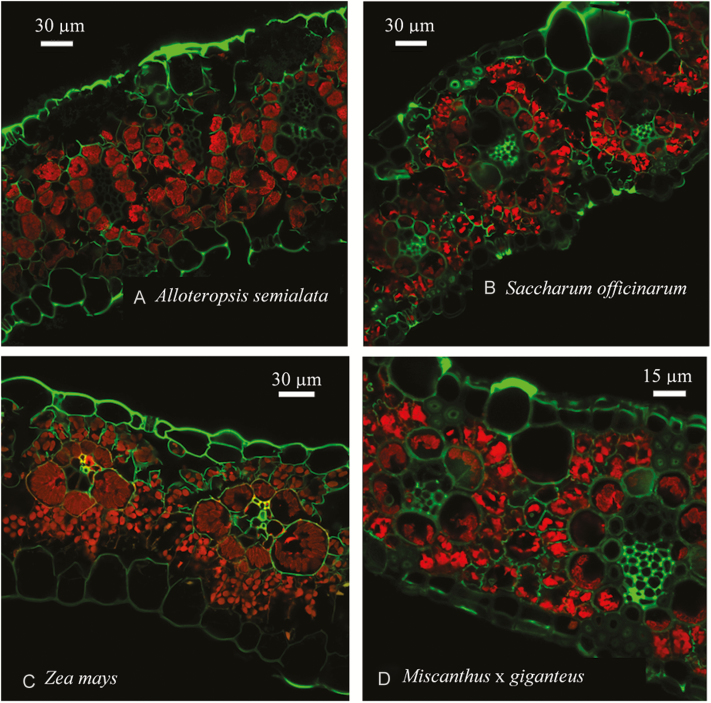
Individual single depth slices of representative leaf cross-sections. Cell walls labeled with FM 1-43FX are green. Chlorophyll fluorescence is red. The darker red bundle sheath fluorescence of *Saccharum officinarum* L., *Zea mays* L. and *Miscanthus* × *giganteus* Greef et Deu. reflects the lower content in the chloroplasts of photosystem II, which is the primary emitter of chlorophyll fluorescence in the detection bandpass of 630–700 nm. The full 3D image of the *Z. mays* leaf is given in Supplementary Video S1.

Supplementary Video S1 at *JXB* online illustrates how the delineation of BS and mesophyll compartments, and the chloroplasts within them, was achieved within a 3D optical section. BS and mesophyll compartments were identified from the fluorescence of dye-labelled cell walls, using image segmentation software (IMARIS 7.0.0 software, BitPlane, Inc., Zürich, Switzerland). These segments were used to determine the volume of BS (vol_BS_) and mesophyll (vol_M_) per unit leaf area. The chlorophyll fluorescence signal within the BS and mesophyll was then used to determine total chloroplast volume per unit leaf area within each compartment (vol_BS,cp_ and vol_M,cp_, respectively) and the percentage occupancy of each compartment by chloroplasts (%_BS,cp_ and %_M,cp_, respectively). Although chlorophyll fluorescence from out-of-focus planes was typically visible in individual optical slices, the surface-finding algorithm of the image segmentation software was able to accurately delineate chloroplast volumes when processing the overall 3D optical section. As a result, individual 2D slices appear to overestimate chloroplast content of cells, but the 3D sections actually used to produce measurements do not; this can be seen by comparing Fig. 1C with Supplementary Video S1.

Leaf thickness was measured in a single location per image, across the mesophyll between two veins, and inter-veinal distance (IVD) was measured as the average distance between the centers of all the adjacent vascular bundles visible in each image.

### Calculating photosynthetic capacity

An important goal of this study was to determine the theoretical maximum amount of Rubisco that C_4_ BS chloroplasts could contain, in order to calculate the corresponding theoretical maximum level of Rubisco-limited photosynthetic CO_2_ uptake (*A*_max,cp_) that could be achieved by a given leaf. Calculated values for *A*_max,cp_ could then be compared to achieved values for light-saturated photosynthetic CO_2_ uptake (*A*_sat_). Because *A*_max,cp_ is a measure of theoretical, and not achieved, Rubisco-limited CO_2_ uptake, factors such as leaf N content and incident light intensity could be ignored. Instead, *A*_max,cp_ was determined from the volume of BS chloroplasts available for Rubisco investment (vol_BS,cp_), the amount of Rubisco that could be contained within these chloroplasts, and the carboxylation activity of Rubisco. Although there is evidence of C_4_ subspecies of *A. semialata* expressing Rubisco in chloroplasts outside of the BS (Ueno and Sentoku, 2006), here it was assumed in all species that only BS chloroplasts contained Rubisco.

vol_BS,cp_ was determined experimentally in this study as described above. A Rubisco carboxylation rate per site at 25 °C (*k*_cat_) of 3.3 mol CO_2_ mol site^−1^ s^−1^ had been determined previously for both *Z. mays* and *M.* × *giganteus* (Wang *et al.*, 2008*a*). This value was reduced by 15%, reflecting the Rubisco activation state at 25 °C of 85%, reported for *M.* × *giganteus* (Wang *et al.*, 2008*a*). This gives an estimated carboxylation rate of 41.6 µmol CO_2_ g^−1^ Rubisco s^−1^ at 25 °C. Rubisco content per unit chloroplast volume was assumed to be 2.2 × 10^5^ g Rubisco m^−3^ chloroplast based on measurements for mesophyll chloroplasts of several genotypes of the hexaploid bread wheat *Triticum aestivum* L. (Pyke and Leech, 1987). Combining the carboxylation rate per gram Rubisco calculated with a molecular mass of 540 kDA, with the grams of Rubisco per unit volume of chloroplast, leads to a theoretical maximal photosynthetic rate of 9.2 mol CO_2_ m^−3^ chloroplast s^−1^ at 25 °C. In the Results, this factor is combined with measured BS chloroplast volume (vol_BS,cp_) to determine the potential photosynthetic rate that could theoretically be supported given the measured chloroplast volume (*A*_max,cp_).

To extend this estimation to temperatures below 25 °C, an Arrhenius function was used based on the activation energy (*E*_a_) of 78 kJ mol^−1^ determined for Rubisco in the C_4_ grass *Setaria viridis* (L.) P.Beauv. (Boyd *et al.*, 2015). To compare this estimation with achieved photosynthesis values, the literature was reviewed to identify values for light-saturated net leaf CO_2_ uptake (*A*_sat_) at moderate and chilling temperatures in all four species: *Z. mays* (Long, 1983; Naidu *et al.*, 2003; Naidu and Long, 2004; Głowacka *et al.*, 2016), *S. officinarum* (Spitz, 2015; Głowacka *et al.*, 2016), *A. semialata* (Osborne *et al.*, 2008), and *M.* × *giganteus* (Naidu *et al.*, 2003; Naidu and Long, 2004; Głowacka *et al.*, 2014, 2015, 2016; Spitz, 2015; Friesen and Sage, 2016), using values measured at different temperatures and at a photon flux ≥1000 μmol m^−2^ s^−1^.

### Statistical analysis

Replication was: *Z. mays* (*n*=7), *S. officinarum* (*n*=5), *A. semialata* (*n*=6), and *M.* × *giganteus* (*n*=6). Statistical analysis was performed on the following parameters: leaf thickness, IVD, vol_BS_, vol_M_, vol_BS,cp_, vol_M,cp_, %_BS,cp_, and %_M,cp_. The fixed effect of species on each parameter was tested by one-way ANOVA (PROC GLM, SAS v8.02; SAS Institute Inc., Cary, NC, USA), with homogeneity of variances tested by Levene and normality of residuals tested by Shapiro–Wilk (PROC UNIVARIATE, SAS v8.02) at a *P*=0.05 threshold. A Tukey test was performed alongside the ANOVA at a *P*=0.05 threshold in order to identify significant pairwise differences between species. When no significant differences were found, the test was repeated at a *P*=0.1 threshold to reduce the risk of a type II error given the relatively low replication for each species.

## Results

**Fig. 2.**
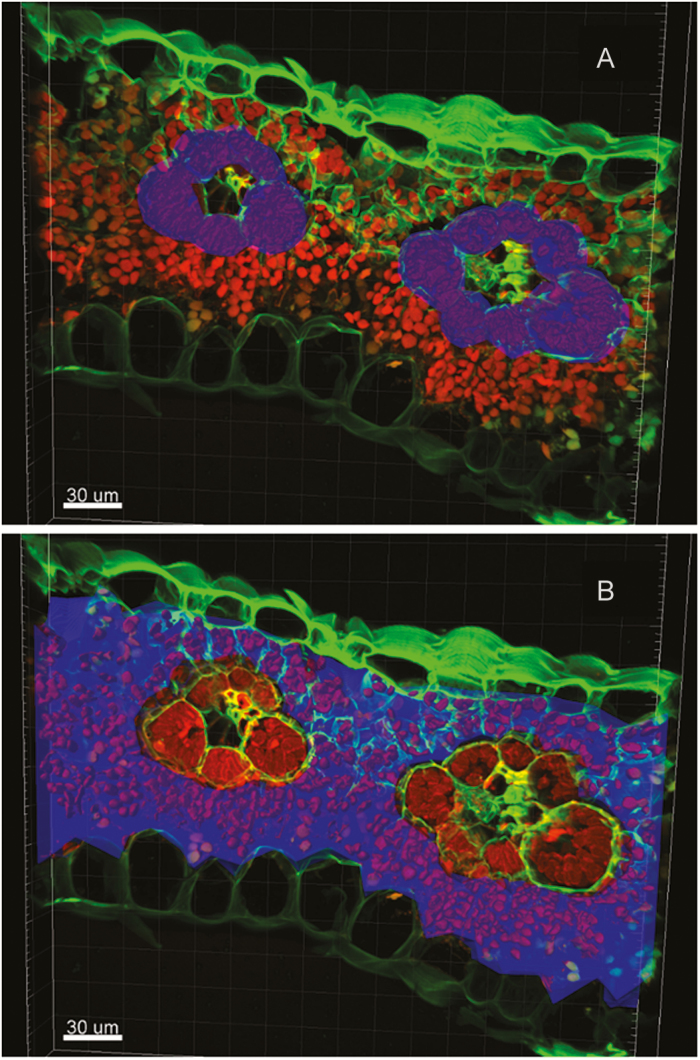
Fluorescence image of a representative *Zea mays* L. leaf. 2D compression of a 3D cross-section of *Z. mays*, 300 µm in length and 15 µm in depth. The full 3D image is given in Supplementary Video S1. Cell walls labeled with FM 1-43FX are green. Chlorophyll fluorescence is red. Delineated volume reconstruction of the bundle sheath and mesophyll compartments is shown in blue in (A) and (B), respectively. Chlorophyll fluorescence was used by the software to reconstruct chloroplast volumes within the bundle sheath and mesophyll; these are shown in bold red in (A) and (B), respectively.

**Fig. 3.**
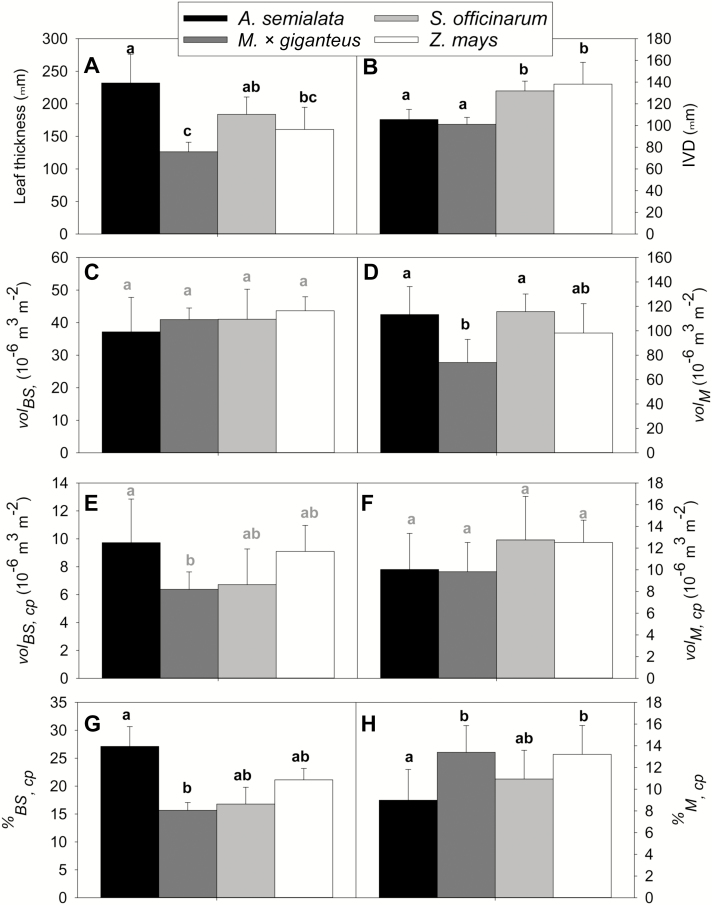
Leaf anatomical characteristics and differences between the study-species. Mean + SE of leaf thickness (A), inter-veinal distance (IVD) (B), bundle sheath volume per leaf area (vol_BS_) (C), mesophyll volume per leaf area (vol_M_) (D), bundle sheath chloroplast volume per leaf area (vol_BS,cp_) (E), mesophyll chloroplast volume per leaf area (vol_M,cp_) (F), occupancy of the bundle sheath by chloroplasts (%_BS,cp_) (G), and occupancy of the mesophyll by chloroplasts (%_M,cp_) (H) in *Zea mays* L. (*n*=7), *Saccharum officinarum* L. (*n*=5), *Alloteropsis semialata* J. Presl (*n*=6), and *Miscanthus* × *giganteus* Greef et Deu. (*n*=6). Lowercase letters indicate Tukey groups, with black letters indicating significant difference at *P*<0.05 and grey letters indicating significant difference at *P*<0.1.

The average volume of chloroplasts per unit leaf area ranged from 6 × 10^–6^ to 10 × 10^–6^ m^3^ m^−2^ in the BS and from 10 × 10^–6^ to 14 × 10^–6^ m^3^ m^−2^ in the mesophyll (Figs 1, 2, 3E, F). There was no evidence of greater BS chloroplast volume available per unit leaf area (vol_BS,cp_) in the chilling-tolerant *M.* × *giganteus* compared with the chilling-sensitive species. On the contrary, *M.* × *giganteus* had the smallest BS chloroplast volume per unit leaf area, at *ca*. 40% less than the wild and chilling-sensitive *A. semialata*. Although there were no significant differences between species in vol_BS_, significantly greater occupancy of the BS by chloroplasts (%_BS,cp_) resulted in greater vol_BS,cp_ overall in *A. semialata* (Fig. 3C, E, G).

Across the four study-species, chloroplasts occupied 15–30% of the BS (%_BS,cp_), and 8–14% of the mesophyll (%_M,cp_) (Figs 1, 3G, H; Supplementary Video S1). Between species, %_BS,cp_ and %_M,cp_ were significantly highest and lowest, respectively, in *A. semialata*. Leaf thickness ranged from 100 to 250 µm, with veins spaced 100–140 µm apart on average (Figs 1, 3A, B). *Alloteropsis semialata* leaves at *ca*. 225 µm were nearly twice as thick as those of *M*. × *giganteus* at *ca*. 125 µm. The distance between veins (IVD) in the two crops (*Z. mays* and *S. officinarum*) was *ca*. 40% greater than in the two wild species (*M.* × *giganteus* and *A. semialata*) (Fig. 3B). Across the species, the volume of mesophyll per unit leaf area (vol_M_) generally mirrored leaf thickness, though due to a thick epidermis the significantly greater leaf thickness of *A. semialata* did not result in a substantially greater vol_M_ (Fig. 3D). BS volume per unit leaf area (vol_BS_), however, was conserved across species at *ca*. 40 × 10^–6^ m^3^ m^−2^ (Fig. 3C).

**Fig. 4.**
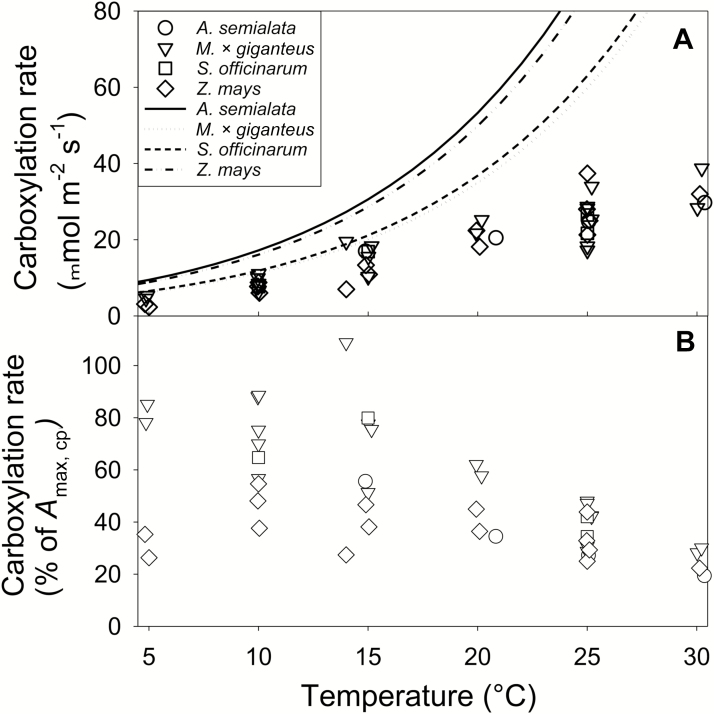
Comparison of theoretical maximum versus achieved leaf photosynthetic carboxylation rates at different temperatures. (A) Symbols indicate published rates of net CO_2_ uptake (*A*_sat_) measured on leaves at different temperatures. Lines show estimated leaf maximal photosynthetic capacity (*A*_max,cp_) calculated from bundle sheath chloroplast volume per unit leaf area. (B) Measurements of *A*_sat_ expressed as a percentage of *A*_max,cp_. Measurements were obtained for *Zea mays* L. (Long, 1983; Naidu *et al.*, 2003; Naidu and Long, 2004; Głowacka *et al.*, 2016), *Saccharum officinarum* L. (Spitz, 2015; Głowacka *et al.*, 2016), *Alloteropsis semialata* J. Presl (Osborne *et al.*, 2008), and *Miscanthus* × *giganteus* Greef et Deu. (Naidu *et al.*, 2003; Naidu and Long, 2004; Głowacka *et al.*, 2014, 2015, 2016; Spitz, 2015; Friesen and Sage, 2016) at different temperatures and at an incident photon flux ≥1000 μmol m^−2^ s^−1^.

When the maximal theoretical photosynthetic capacity of the leaf (*A*_max,cp_) was estimated from vol_BS,cp_, values ranged from *ca*. 60 to 90 µmol m^−2^ s^−1^ at 25 °C. This was substantially greater than published values of light-saturated net photosynthetic CO_2_ uptake (*A*_sat_) for these species at this temperature (Fig. 4). However, at lower temperatures *A*_sat_ was closer to *A*_max,cp_, with *A*_sat_ being 20–90% of *A*_max,cp_ at 5 °C.

## Discussion

In all four of the C_4_ grass species studied here, the volume of BS per unit leaf area available for Rubisco (vol_BS_) was not a limitation for observed rates of photosynthesis, even at chilling temperatures. This conclusion is based on two key findings, derived from 3D confocal microscopy and analysis of leaf structure (Fig. 2). First, the chilling-tolerant *M.* × *giganteus* (Long and Spence, 2013) has a smaller BS chloroplast volume per unit leaf area (vol_BS,cp_) than the chilling-sensitive C_4_ grasses *S. officinarum*, *A. semialata*, and *Z. mays* (Fig. 3). Second, the theoretical maximum level of Rubisco-limited photosynthetic CO_2_ uptake (*A*_max,cp_) that could be achieved by each species was greater than realized levels of *A*_sat_, even at chilling temperatures (Fig. 4). This study focused on closely related C_4_ grasses of the Andropogoneae clade, which contain the major C_4_ crops as well as candidate bioenergy crops. Even *A. semialata*, which descends from a separate evolutionary lineage in the Paniceae, would not suffer from limitation of photosynthesis by vol_BS_ during chilling.

Several leaf structural characteristics, including leaf thickness, IVD, vol_M_, %_BS,cp_, and %_M,cp_, varied significantly between species (Figs 1, 3A, B, D, G, H). Indeed, the vol_BS,cp_ was actually greatest in the chilling-sensitive *A. semialata* and lowest in the chilling-tolerant *M.* × *giganteus* (Fig. 3E). This clearly demonstrates that vol_BS,cp_ does not determine chilling tolerance in C_4_ plants, and therefore that the volume of BS chloroplast available for leaf Rubisco investment is unlikely to meaningfully restrict C_4_ photosynthesis at low temperatures.

Based on 2D leaf profiles, the percentage occupancy of the total mesophyll volume by chloroplasts varies significantly between photosynthetic types and taxonomic clades of diverse C_4_ plants, with an average occupation of *ca*. 12.2% (Stata *et al.*, 2014), which is similar to the 8–14% range seen here (Figs 1, 3H). In various species of the eudicot genus *Flaveria* that use the NADP-ME subtype of C_4_ photosynthesis, chloroplasts occupied 12–18% of the total BS volume (Stata *et al.*, 2016), which is somewhat lower than the range of 15–25% seen in our grasses (Figs 1, 3G); this may reflect differences due to taxonomy or specimen preparation. *Alloteropsis semialata*, which belongs to the Paniceae tribe, had the greatest volume of chloroplast in the BS (%_BS,cp_) (Figs 1, 3G, H). This may reflect this species’ need to house grana in their BS chloroplasts, while the other three studied grasses of the Andropogoneae tribe have little to no BS chloroplast grana (Ueno and Sentoku, 2006). *Alloteropsis semialata*’s high BS chloroplast volume may also result from the very recent development of C_4_ anatomy in this species, which might not have evolved the faster Rubisco kinetics of other, older C_4_ lineages and could therefore require relatively more Rubisco in the BS to compensate (Lundgren *et al.*, 2015; Dunning *et al.*, 2017).

While chloroplasts across the entire mesophyll tissue are available for Rubisco investment in C_3_ plants, there is clearly less space available in the BS tissue of C_4_ leaves. However, in the mesophyll of C_3_ species, CO_2_ must diffuse from the air space to Rubisco in the chloroplast, and chloroplasts must be adjacent to the cell wall to maximize mesophyll conductance to CO_2_ and facilitate access of Rubisco to CO_2_ (Evans and Loreto, 2000; Flexas *et al.*, 2008). In the BS of C_4_ species, CO_2_ results from decarboxylation of C_4_-dicarboxylates in the chloroplast or the cytosol, and the effective chloroplast volume is therefore not limited by the area of wall adjacent to air space. In effect, this can allow larger and more numerous chloroplasts, and may explain the greater proportion of the BS cell occupied by chloroplasts, relative to mesophyll (Figs 1, 3G, H).

The comparison of *A*_max,cp_ to published values for *A*_sat_ is directly dependent on terms used to calculate *A*_max,cp_: for instance, a 20% lower value for *k*_cat_ will result in 20% lower *A*_max,cp_. At lower temperatures this could lead to *A*_max,cp_ much closer to published values for *A*_sat_ (Fig. 4A, B). However, the values used in this study were generally conservative. In a survey of Rubisco *k*_cat_ in 14 grasses using different subtypes of C_4_ photosynthesis (Ghannoum *et al.*, 2005), all seven NADP-ME grasses and five of the seven NAD-ME grasses registered values greater than, and up to two times, the *k*_cat_ value used here; i.e. 3.3 mol CO_2_ mol site^−1^ s^−1^ (Wang *et al.*, 2008*a*).

Another important term in the calculation of *A*_max,cp_ is the Rubisco content per unit volume chloroplast. Here, we used a published value of 0.41 mol Rubisco m^−3^ chloroplast, derived from *T. aestivum* mesophyll chloroplasts (Pyke and Leech, 1987). This value is conservative, as it is at the lower end of the 0.4–0.5 mol Rubisco m^−3^ chloroplast range predicted from measurements in C_3_ chloroplasts (Jensen and Bahr, 1977). Furthermore, C_4_ plants generally produce larger chloroplasts than C_3_ plants, particularly in the BS (Brown and Hattersley, 1989; Stata *et al.*, 2014) and these chloroplasts likely contain more Rubisco per unit volume, since NADP-ME C_4_ grasses, including *Z. mays*, *S. officinarum*, and *M.* × *giganteus*, typically show few or no stacked thylakoids in the BS. This arrangement leaves more space available for stroma, and therefore Rubisco, in comparison with bread wheat chloroplasts (Furbank, 2011; Voznesenskaya *et al.*, 2006, 2007; Bellasio and Griffiths, 2014).

Despite the use of conservative terms to calculate *A*_max,cp_, this parameter was greater than published light-saturated photosynthetic rates (*A*_sat_) for all four studied species (Fig. 4) (Long, 1983; Naidu *et al.*, 2003; Naidu and Long, 2004; Osborne *et al.*, 2008; Głowacka *et al.*, 2014, 2015, 2016; Spitz, 2015; Friesen and Sage, 2016). This was even true at low temperatures, where Rubisco has been predicted to be a strong limitation to C_4_ photosynthesis (Pearcy, 1977; Pittermann and Sage, 2000; Kubien *et al.*, 2003; Kubien and Sage, 2004). Therefore, we conclude that while the quantity of Rubisco may be limiting, this is not an inherent result of the smaller proportion of cells that can contain the enzyme in C_4_ leaves with Kranz anatomy. Further supporting our conclusion that BS chloroplast space does not limit Rubisco comes from the fact that Rubisco content does increase in *M.* × *giganteus* on chilling (Long and Spence, 2013). Additional evidence comes from a recent transgenic up-regulation of Rubisco content by >30% above wild type in leaves of *Z. mays* (Salesse *et al.*, 2018).

Based on genetic diversity, the assumed origin of the C_4_ grass tribe Andropogoneae is tropical Southeast Asia (Hartley, 1958; Arthan *et al.*, 2017). Tropical origins are common across the C_4_ grass clades (Watcharamongkol *et al.*, 2018). Radiation into temperate climates has therefore involved solving the challenges of chilling and freezing temperatures faced by all tropical plants, regardless of photosynthetic type, as well as any additional restrictions added by the C_4_ cycle and associated anatomy. The literature has already addressed these additional restrictions and the evolution of chilling-tolerant C_4_ photosynthesis (Long, 1983, 1999; Long and Spence, 2013).

Several C_4_ grasses, including *Muhlenbergia glomerata* (Kubien and Sage, 2004), *Spartina anglica* (Long *et al.*, 1975), and *Cleistogenes squarrosa* (Liu and Osborne, 2008) can achieve rates of CO_2_ assimilation at chilling temperatures that equal or exceed rates achieved by temperate and even arctic/alpine C_3_ grasses. Notably, the C_4_ grass *M. × giganteus* appears exceptional in its ability to acclimate its photosynthetic apparatus to chilling temperatures. Comparison with the chilling-intolerant *Z. mays* suggests that chilling tolerance in *M.* × *giganteus* results from its ability to maintain and increase the expression of the enzymes PPDK and Rubisco, as well as increase leaf xanthophyll content, in particular zeaxanthin, to harmlessly dissipate excess absorbed light energy under chilling conditions and protect photosystem II from oxidative damage (reviewed in Long and Spence, 2013). Gene expression analyses suggest that these increases are part of a syndrome of acclimative changes that allow efficient C_4_ photosynthesis under chilling conditions (Spence *et al.*, 2014), and in turn the exceptional productivities achieved by *M.* × *giganteus* in temperate climates (Dohleman and Long, 2009). Therefore, while Rubisco content clearly co-limits photosynthesis in many C_4_ species under chilling conditions, the findings here show that this does not directly result from restricting Rubisco to the BS in C_4_ grasses.

In conclusion, while the volume of the cells that can hold Rubisco in C_4_ grass leaves is lower than in their C_3_ counterparts, measurements of BS chloroplast volume show that space *per se* does not present a physical, and in turn intrinsic, limitation on photosynthesis at chilling temperatures. Therefore, restriction of leaf Rubisco content by the volume of BS chloroplasts does not inherently limit the adaptation of C_4_ grasses to cold environments.

## Supplementary data

Video S1. Video of the full 3D image of leaf, bundle sheath cells, mesophyll cells, and chloroplasts seen in Fig. 2.

Supplementary Video S1

## Abbreviations:

*A*_max,cp_: *A*_sat_ that could be supported by the Rubisco that could be accommodated in theory within the measured bundle sheath chloroplast volume (µmol m^−2^ s^−1^)
*A*_sat_: light-saturated net rate of photosynthetic CO_2_ assimilation in leaves (µmol m^−2^ s^−1^)
BS: bundle sheath
IVD: inter-veinal distance (µm)
vol_BS_: bundle sheath volume per unit leaf area (m^3^ m^−2^)
vol_BS,cp_: bundle sheath chloroplast volume per unit leaf area (m^3^ m^−2^)
vol_M_: mesophyll volume per unit leaf area (m^3^ m^−2^)
vol_M,cp_: mesophyll chloroplast volume per unit leaf area (m^3^ m^−2^)
%_BS,cp_: percentage occupancy of the bundle sheath by chloroplasts (dimensionless)
%_M,cp_: percentage occupancy of the mesophyll by chloroplasts (dimensionless)
